# Perceived ease of flavored e-cigarette use and e-cigarette use progression among youth never tobacco users

**DOI:** 10.1371/journal.pone.0212353

**Published:** 2019-02-27

**Authors:** Julia Cen Chen-Sankey, Grace Kong, Kelvin Choi

**Affiliations:** 1 Division of Intramural Research, National Institute on Minority Health and Health Disparities, Bethesda, MD, United States of America; 2 Department of Psychiatry, Yale University School of Medicine, New Haven, CT, United States of America; Brown University, UNITED STATES

## Abstract

**Background:**

There is an increased need to understand how e-cigarette flavors may contribute to e-cigarette uptake and use among youth. We examined the relationship between perceived ease of flavored e-cigarette use and e-cigarette use susceptibility and progression among a nationally representative sample of U.S. youth never tobacco users.

**Methods:**

The wave 1 (2013–2014) and wave 2 (2014–2015) surveys of PATH Study were used. Youth never tobacco users (ages 12–17) who reported whether flavored e-cigarettes were easier to use than unflavored e-cigarettes at wave 1 (n = 6,983) were included in the study. Multivariable logistic regressions were used to examine the associations between perceived ease of using flavored e-cigarettes (wave 1) and e-cigarette use outcomes including e-cigarette use susceptibility (wave 1) and e-cigarette initiation and past-30-day use (wave 2).

**Results:**

Overall, 21.2% of the sample perceived flavored e-cigarettes easier to use than unflavored e-cigarettes; and 28.9% of the sample were susceptible to using e-cigarettes at wave 1, and 7.5% and 2.0% initiated e-cigarettes and used e-cigarettes in the past 30 days at wave 2, respectively. Among those who perceived flavored e-cigarettes easier to use, 41.0% were susceptible to using e-cigarettes at wave 1, and 10.6% and 3.4% initiated and used e-cigarettes in the past 30 days at wave 2, respectively. Perceiving flavored e-cigarettes as easier to use than unflavored e-cigarettes at wave 1 was positively associated with e-cigarette use susceptibility at wave 1 (AOR = 1.43, CI = 1.21, 1.69), and e-cigarette initiation (AOR = 1.32, CI = 1.12, 1.67) and past-30-day use (AOR = 1.25, CI = 1.10, 2.47) at wave 2.

**Conclusions:**

Perceiving flavored e-cigarettes as easier to use than unflavored e-cigarettes may lead to e-cigarette use progression among youth never tobacco users. Determining the factors (including e-cigarette marketing and specific e-cigarette flavors) that lead to perceived ease of using flavored e-cigarettes would inform efforts to prevent and curb youth e-cigarette use.

## Introduction

E-cigarette use among youth has risen in the past few years. In 2016, 4.3% and 11.3% of U.S. middle and high school students reported using e-cigarettes in the past month, increasing from 0.6% and 1.5% in 2011, respectively [1]. One potential reason for their popularity is that e-cigarettes come in a variety of flavors that are appealing to youth [2]. As of 2013, more than 7,000 flavors of e-cigarettes have been marketed in the US [3], and many of these flavors are especially popular among youth [4,5]. For instance, national data indicate that about 85.0% of youth who used e-cigarettes in the past 30 days adopted non-tobacco flavors such as fruit, candy, and dessert [5].

E-cigarette devices and e-liquid constituents, including flavorings, could have negative health consequences among youth and especially youth never tobacco users. E-cigarettes can contain nicotine, and nicotine exposure during adolescence can have adverse consequences on long-term cognitive and behavioral impairments [6]. E-cigarette users have previously reported adverse health symptoms such as mouth and throat irritation, cough, and headache [7]. Particularly, e-cigarette use among youth with no or little tobacco use history may lead to a greater intention of smoking cigarettes as well as a greater likelihood of future cigarette use [8,9] and multiple combustible tobacco use [10]. E-cigarettes with attractive flavors may further entice youth to experiment with e-cigarettes [11] and boost e-cigarettes’ influence on increased cigarette smoking susceptibility among youth [12]. The 2017 Surgeon General Report concluded that due to the negative health consequences of using e-cigarettes among youth, e-cigarette use among this group should be prevented and minimized [13]. Moreover, restricting or eliminating e-cigarette flavors may prevent or reduce e-cigarette use initiation and regular use among the youth population. For example, a previous study indicates that the national sales restriction of flavored cigarettes in 2009 was associated with a 17% reduction in cigarette smoking prevalence and a 58% decrease in cigarette consumption among youth [14].

Current evidence on youth perceptions of flavored e-cigarettes focuses on youth’s preferences for and attraction to flavored e-cigarettes [15], flavors as the main reason of using e-cigarettes [16,17], as well as the reduced harm perceptions about flavored e-cigarettes as compared to unflavored e-cigarettes [18]. However, little is known about the perceived benefits of using flavored e-cigarettes among this group and whether this perception leads to youth e-cigarette use intentions and behaviors. According to the Health Belief Model [19], perceived benefits of conducting a health behavior predicts the likelihood of engaging in the behavior. One important perceived benefit is the perceived ease of using flavored e-cigarettes. Youth may perceive flavored e-cigarettes easy to use due to the wide availability of flavored e-cigarette products and the familiar and enjoyable sensory experience that e-cigarette flavors produce. With the growing popularity of e-cigarette pod systems (e.g., JUUL) which offer a wide range of youth-friendly flavors and enable discreet use, e-cigarette products may be increasingly considered easier to use at school and home than ever before among the youth population.

Additionally, understanding whether youth considers flavored e-cigarettes easy to use will contribute to regulatory decisions on flavored e-cigarette products. In March 2018, the U.S. Food and Drug Administration (FDA) issued an Advance Notice of Proposed Rulemakings (ANPRM) [20] to seek public comments about the role of flavors (other than tobacco flavors) on the initiation and continued use of tobacco products. In November 2018, the FDA proposed rules to restrict the sales of flavored e-cigarettes in gas stations and convenience stores in order to prevent youth use of e-cigarettes [21]. This study will further contribute to tobacco regulatory decision-making in regards to flavored e-cigarette products. Due to the pervasiveness of e-cigarette flavors and their popularity among youth, we hypothesized that youth’s perceptions of the relative ease of using flavored e-cigarettes compared to unflavored e-cigarettes may positively influence e-cigarette use susceptibility (self-rated interest in trying e-cigarettes) and progression (initiation and past-30-day use). The goal of this one-year prospective study using secondary data from the Population Assessment of Tobacco and Health (PATH) Study was to examine whether the perceived ease of using flavored e-cigarettes among never tobacco (including e-cigarettes) users at baseline was associated with (1) e-cigarette use susceptibility at baseline, (2) e-cigarette use initiation at follow-up, and (3) past-30-day e-cigarette use at follow-up.

## Methods

### Sample

This study used the wave 1 and 2 surveys from the PATH Study, which is a nationally representative, longitudinal cohort study of U.S. adults and youth. Information on tobacco use and health status among civilian, non-institutionalized individuals was gathered from audio computer-assisted self-interviews in English and Spanish [22]. The wave 1 and 2 surveys were collected during 2013–2014 and 2014–2015, respectively. In this prospective analysis, the sample (n = 6,983) was restricted to youth (ages 12–17 at wave 1) who had answered the wave 1 question about the perceived ease of using flavored e-cigarettes and had never used e-cigarettes or any other tobacco products (i.e., cigarettes, cigar products, hookah, pipe, smokeless tobacco and snus, dissolvable tobacco, and bidis and kreteks) before wave 1. Since the 17-year-old youth at wave 1 aged out of the youth survey at wave 2, wave 2 adult survey data were also used for analysis. The retention rate for waves 1 and 2 youth surveys were 73.8% [23].

### Measures

*Perceived Ease of Using Flavored E-cigarettes* (wave 1) was measured by the question “Are flavored e-cigarettes easier to use, about the same, or harder to use than unflavored e-cigarettes?” The answers included “Easier to use,” “About the same,” “Harder to use,” and “I don’t know.”

E-cigarette use-related variables consisted of *E-cigarette Use Susceptibility* (wave 1), *E-cigarette Initiation* (wave 2), and *Past-30-day E-cigarette Use* (wave 2). E-cigarette Use Susceptibility was measured by three questions: “Have you ever been curious about using e-cigarettes?” “Do you think you will try an e-cigarette soon?” and “If one of your best friends were to offer you an e-cigarette, would you use it?” Each item featured 4 Likert-type responses ranging from “very curious/definitely yes” to “not at all curious/definitely not.” Responses other than “not at all curious/definitely not” for any of the three items was categorized as e-cigarette use susceptibility [24]. The scale demonstrated strong reliability and validity in the youth population [24]. At wave 2, the respondents were asked “Have you ever used an electronic nicotine product, even one or two times? (Electronic nicotine products include e-cigarettes, e-cigars, e-pipes, e-hookah, personal vaporizers, vape pens, and hookah pens).” Those who indicated that they had used an electronic nicotine product before were further asked which specific product including “e-cigarette (vape pens and personal vaporizers)” they had used. Those who responded “Yes” to the “e-cigarette” option were considered as having initiated e-cigarettes between two waves. Past-30-day E-cigarette Use was measured by the question “When was the last time you used an e-cigarette, even one or two times?” with the answer options ranging from “earlier today” to “5 or more years ago.”

All the following covariates were from the youth wave 1 survey of the PATH Study. Demographic covariates used in this study were *age*, *gender*, *race/ethnicity*, and *parent education* (see categories in Table 1). *Perceived harm of using e-cigarettes* was measured by the question “How much do you think people harm themselves when they use e-cigarettes?” Categories for this variable were no/little, some harm, and a lot of harm. Psychosocial covariates used in the study were *sensation seeking*, *lifetime internalized problems*, and *lifetime externalized problems*. Sensation seeking, a risk factor for youth tobacco use [25], was measured by three modified items from the Brief Sensation Seeking Scale (BSSS) in the PATH Study: (1) “I like to do frightening things,” (2) “I like new and exciting experiences even if I have to break the rules,” and (3) “I prefer friends who are exciting and unpredictable.” [26] Response options were: 0 = strongly disagree, 1 = disagree, 2 = neither agree nor disagree, 3 = agree, and 4 = strongly agree, and they were summed to create a mean score (0–4). BSSS demonstrated strong reliability and validity among adolescents [26]. Internalized problems and externalized problems related to mental health were also found to be associated with youth tobacco use [27]. The PATH study modified and included 11 items from the Global Appraisal of Individual Needs–Short Screener (GAIN-SS) [28]. An example item of the four items of internalized problems is “Feeling very trapped/sad/depressed,” and an example item of the seven externalized problem items is “Having hard time paying attention.” The overall scale and sub-scales of GAIN-SS demonstrated moderate to strong reliability in the youth population [27]. For each item, the respondents were asked to choose from four time-periods: never, past month, 2–12 months ago, and over a year ago. The number of responses endorsed in the lifetime was summed to create variables *Lifetime Internalized Problems* and *Lifetime Externalized Problems* that ranged from 0–4 for internalizing problems and 0–7 for externalizing problems [27]. Respondents were categorized into three levels according to their number of lifetime problems: no/low (0–1 problem), moderate (2–3 problems), or high (4 for internalizing problems, or ≥4 symptoms for externalizing problems) [29].

**Table 1:** Demographic and psychosocial covariates, perceived ease of using flavored e-cigarettes compared to unflavored e-cigarettes, and e-cigarette use outcomes—PATH Study youth (aged 12–17) wave 1 and 2 surveys (n = 6,983).

		Perceived Ease of Using Flavored E-cigarettes Relative to Unflavored E-cigarettes								
		Flavored E-cigs Easier		About Same		Flavored E-cigs Harder		Don’t Know		
		%	[95% CI]	%	[95% CI]	%	[95% CI]	%	[95% CI]	P value
Age									<0.0001
	12–14	18.0%	[16.6, 19.4]	43.6%	[41.8, 45.4]	4.4%	[3.9, 5.0]	34.0%	[32.6, 35.5]	
	15–17	26.7%	[24.8, 28.6]	42.3%	[40.2, 44.4]	3.7%	[3.0, 4.5]	27.4%	[25.6, 29.3]	
Gender										<0.0001
	Male	19.4%	[17.9, 20.9]	41.5%	[39.6, 43.4]	4.0%	[3.4, 4.7]	35.1%	[33.5, 36.8]	
	Female	23.0%	[21.3, 24.8]	44.8%	[42.7, 46.9]	4.3%	[3.6, 5.1]	28.0%	[26.5, 29.5]	
Race/Ethnicity										<0.0001
	NH White	19.1%	[17.6, 20.6]	42.1%	[40.1, 44.1]	3.2%	[2.6, 3.9]	35.6%	[33.9, 37.5]	
	NH Black	26.8%	[23.4, 30.5]	42.7%	[38.7, 46.7]	5.9%	[4.6, 7.5]	24.6%	[21.7, 27.8]	
	Hispanic	22.1%	[20.2, 24.1]	46.8%	[44.2, 49.3]	5.9%	[5.0, 6.9]	25.3%	[23.5, 27.2]	
	NH Other	23.2%	[18.6, 28.4]	42.0%	[37.6, 46.5]	3.1%	[1.9, 4.9]	31.8%	[28.0, 35.9]	
Parent Education										<0.0001
	≤HS	21.6%	[19.8, 23.5]	46.9%	[44.5, 49.4]	5.3%	[4.5, 6.2]	26.2%	[24.4, 28.1]	
	Some College	21.6%	[19.7, 23.5]	42.9%	[40.2, 45.5]	4.3%	[3.7, 5.1]	31.3%	[29.3, 33.3]	
	≥College	20.3%	[18.3, 22.4]	39.9%	[37.5, 42.3]	2.8%	[2.1, 3.6]	37.0%	[34.7, 39.4]	
Perceived Harm of Using E-cigarettes										<0.0001
	No/little Harm	28.0%	[25.7, 30.6]	44.1%	[41.3, 46.9]	2.9%	[2.2, 3.7]	25.0%	[22.9, 27.3]	
	Some Harm	21.4%	[19.8, 23.0]	45.1%	[43.3, 46.8]	3.4%	[2.8, 4.2]	30.2%	[28.6, 31.8]	
	A lot of Harm	15.7%	[13.9, 17.6]	42.9%	[40.3, 45.5]	7.0%	[5.9, 8.2]	34.5%	[32.1, 36.9]	
Sensation Seeking Score (Mean: 0–4)										<0.0001
	Mean [95% CI]	1.69	[1.63, 1.74]	1.57	[1.53, 1.60]	1.22	[1.11, 1.33]	1.47	[1.42, 1.51]	
Lifetime Internalized Problems										<0.0001
	No/Low	17.1%	[15.6, 18.7]	42.4%	[40.2, 44.7]	5.4%	[4.6, 6.4]	35.1%	[33.2, 37.1]	
	Moderate	21.6%	[19.6, 23.7]	41.5%	[38.8, 44.3]	3.4%	[2.8, 4.1]	33.6%	[31.3, 35.8]	
	High	24.7%	[22.6, 27.0]	44.2%	[41.9, 46.5]	3.4%	[2.7, 4.3]	27.6%	[25.7, 29.8]	
Lifetime Externalized Problems										<0.0001
	No/Low	17.4%	[15.8, 19.1]	42.2%	[40.2, 44.2]	5.3%	[4.5, 6.3]	35.1%	[33.4, 36.9]	
	Moderate	23.5%	[22.1, 25.0]	42.3%	[40.3, 44.3]	3.1%	[2.5, 3.8]	31.2%	[29.5, 32.9]	
	High	25.0%	[21.3, 29.2]	47.2%	[43.0, 51.5]	4.1%	[2.8, 5.9]	23.7%	[20.6, 27.1]	
E-cigarette Use Susceptibility										<0.0001
	Yes	29.6%	[27.4, 31.9]	42.2%	[39.9, 44.5]	3.0%	[2.4, 3.7]	25.2%	[23.6, 27.1]	
	No	17.8%	[16.7, 19.0]	42.9%	[41.3, 44.5]	4.8%	[4.3, 5.5]	34.5%	[33.2, 35.9]	
E-cigarettes Use Initiation										<0.0001
	Yes	30.2%	[26.7, 36.3]	44.2%	[39.6, 48.9]	1.8%	[1.0, 3.3]	23.8%	[20.6, 28.2]	
	No	20.5%	[19.3, 21.8]	41.5%	[40.3, 43.5]	4.3%	[3.8, 4.8]	33.7%	[31.0, 33.6]	
Past-30-day E-cigarette Use										<0.01
	Yes	34.1%	[26.0, 43.2]	42.8%	[34.6, 51.7]	1.8%	[0.4, 7.4]	21.3%	[14.4, 30.3]	
	No	20.8%	[19.6, 22.1]	43.1%	[41.5, 44.7]	4.2%	[3.7, 4.7]	31.9%	[30.7, 33.2]	

### Statistical analysis

We examined the relationships between perceived ease of using flavored e-cigarettes at wave 1 and (1) e-cigarette use susceptibility at wave 1, (2) e-cigarette initiation at wave 2, and (3) past-30-day e-cigarette use at wave 2. We first used chi-square tests and t-tests to explore the associations between perceived ease of using flavored e-cigarettes with the e-cigarette outcome variables, demographics, and psychosocial covariates. We then used three sets of multivariable logistic regressions to explore the associations between perceived ease of using flavored e-cigarettes with each of the three e-cigarette use variables, controlling for covariates. The study’s analysis involved using the Stata 14.0 survey command to account for the wave 2 weights for calculating proportions with 95% confidence intervals, using the balanced repeated replications (BRR) method with Fay’s adjustment (p = 0.3) [23]. The wave 2 weights, which are longitudinal, also account for nonresponse from wave 1 to wave 2 [23]. Imputed demographic variables included in the PATH public data files were used. Listwise deletion was used to handle missing data due to non-responses to specific items. Missing data for each of the three regression models were less than 5% of the entire sample [30]. The significance level of the statistical analysis was set at *p*<0.05. This research is a secondary data analysis of deidentified data and was determined by the National Institutes of Health Office of Health Subjects Research Protection to be exempted from review by an Institutional Review Board.

## Results

Among the youth respondents who had never used tobacco products at wave 1 (N = 6,983), 21.2% perceived the use of flavored e-cigarettes to be easier, 43.1% about the same, and 4.1% more difficult than unflavored e-cigarettes. Additionally, 32.6% of youth reported not knowing whether flavored e-cigarettes were more or less difficult to use relative to unflavored e-cigarettes. Furthermore, 28.9% of the respondents were susceptible to using e-cigarettes at wave 1, 7.5% initiated e-cigarette use at wave 2, and 2.0% used e-cigarettes in the past 30 days at wave 2.

Table 1 presents the associations between demographic and psychosocial characteristics and the perceived ease of using flavored e-cigarettes. Youth aged 15–17 years were more likely to perceive flavored e-cigarettes as easier to use than younger respondents aged 12–14 years (*p*<0.0001). Females were more likely to perceive flavored e-cigarettes as easier to use than males (*p*<0.0001). Non-Hispanic (NH) Black and Hispanic youth were more likely to perceive flavored e-cigarettes as easier to use than their NH White peers (*p*<0.0001). Additionally, youth who had higher sensation seeking scores (*p*<0.0001), had moderate to high lifetime internalized problems (*p*<0.0001), or had moderate to high externalized problems (*p*<0.0001) were more likely to perceive flavored e-cigarettes as easier to use than those who had lower sensation seeking scores or lower levels of internalized and/or externalized problems. The results also showed that youth who were susceptible to using e-cigarettes at wave 1 (*p*<0.0001) were more likely to perceive flavored e-cigarettes as easier to use at wave 1 compared to those who did not have susceptibility. The youth who initiated e-cigarettes at wave 2 (*p*<0.0001) were also more likely to perceive flavored e-cigarettes as easier to use at wave 1 compared to those who did not initiate e-cigarettes. Additionally, the youth who used e-cigarettes in the past 30 days at wave 2 (*p*<0.001) were more likely to perceive flavored e-cigarettes as easier to use at wave 1 compared to those who did not use e-cigarettes in the past 30 days.

Table 2 summarizes the associations between the perceived ease of using flavored e-cigarettes and e-cigarette use variables, controlling for respondents’ demographic and psychosocial characteristics. Using the response of “about the same” as the reference category, the youth who reported flavored e-cigarettes as easier to use than unflavored e-cigarettes were more likely to be susceptible to using e-cigarettes at wave 1 (AOR = 1.43, CI = 1.21, 1.69, *p*<0.001), to have initiated e-cigarette use at wave 2 (AOR = 1.32, CI = 1.12, 1.67, *p*<0.05), and to use e-cigarettes in the past 30 days at wave 2 (AOR = 1.25, CI = 1.10, 2.47, *p*<0.05). Additionally, the respondents who reported “I don’t know” to the perceived ease of use question were less likely to have e-cigarette use susceptibility (AOR = 0.82, CI = 0.69, 0.96, *p*<0.01) at wave 1.

**Table 2:** Adjusted associations between perceived ease of using flavored e-cigarettes and e-cigarette use outcomes—PATH Study youth never tobacco users (aged 12–17) wave 1 and 2 surveys (n = 6,983).

			E-cigarette Use Susceptibility		E-cigarettes Use Initiation		Past-30-day E-cigarette Use	
			%	AOR [95% CI]	%	AOR [95% CI]	%	AOR [95% CI]
Perceived Ease of Using Flavored E-cigarettes vs. Unflavored E-cigarettes								
	Flavored E-cigarettes Easier	41.0%	**1.43 [1.21, 1.69]**	10.6%	**1.32 [1.12, 1.67]**	3.4%	**1.25 [1.10, 2.47]**
	About the Same		29.8%	Reference	8.0%	Reference	2.2%	Reference
	Flavored E-cigarettes Harder	20.2%	0.71 [0.50, 1.03]	3.9%	0.65 [0.33, 1.13]	0.8%	0.59 [0.07, 4.32]
	Don’t Know		24.1%	**0.82 [0.69, 0.96]**	5.9%	0.81 [0.64, 1.19]	1.4%	0.77 [0.47, 1.30]

Note 1: The multivariate regression model adjusted for age, gender, race/ethnicity, parent education, sensation seeking, lifetime internalized problems, lifetime externalized problems, and harm perceptions of using e-cigarettes.

Note 2: Bolded estimates are statistically significant (*p*<0.05).

## Discussion

This is the first nationally representative youth study to provide evidence on the perceived ease of using flavored e-cigarettes and its relationship with e-cigarette susceptibility and future initiation and use. The study found that among youth who had never tried any types of tobacco before, those who perceived flavored e-cigarettes as easier to use than unflavored e-cigarettes at wave 1 were more likely to report e-cigarette use susceptibility at wave 1, initiation of e-cigarette use at wave 2, and past-30-day use of e-cigarettes at wave 2. Evidence has shown that the palatability and appeal of flavors in e-cigarettes serve as one of the main reasons for youth to initiate and regularly use e-cigarettes [31,32]. Thus, it is reasonable to assume that youth who perceive flavored e-cigarettes as easier to use have a higher affinity for e-cigarette products, and thus have an increased tendency to be open to using e-cigarettes and trying e-cigarette products. Having a positive view towards flavored e-cigarettes may facilitate youth’s adoption of flavored e-cigarette products which have been predominantly used by this group [33] and whose market share has increased drastically over the past few years [34].

Given the alarming consequence of perceiving flavored e-cigarettes easy to use among youth never tobacco users, it is important to prevent the development of such perceptions in this group. Thus, it is critical to understand the factors that contribute to the perception. First, youth have strong preferences for sugar and sweet tastes [35] because sugar releases opioids and dopamine and thus might have addictive potential through providing pleasurable sensations to users [36]. Thus, the sensory similarities between sweet foods and e-cigarette flavors (especially sweet flavors) could reduce psychological barriers to using flavored e-cigarettes. Certain e-cigarette flavors (e.g., tobacco, fruit, candy, desserts etc.) that are particularly appealing to youth may also play a significant role in influencing the ease of use perceptions than other flavors. Second, the wide availability of flavored e-cigarettes may increase youth’s perceived access to the product through a variety of channels including social connections, non-compliant tobacco vendors, and online stores that sell e-cigarettes [37]. Although there has been a recent trend of restricting flavored e-cigarettes among U.S. local jurisdictions, these restrictions were found to lack strictness in constraining the availability of e-cigarette flavors (such as leaving menthol flavors exempted) therefore may still leave youth with abundant access to flavored e-cigarettes [38].

Additionally, e-cigarette advertisements may portray flavored e-cigarettes as less harmful than unflavored e-cigarettes, thereby fostering the impression that the product is easier to use. Research studies have found that in 2014, 68.9% of U.S. school-attending youth were exposed to e-cigarette advertisements [39], and that e-cigarette marketing exposure was positively associated with youth’s e-cigarette use [40]. Furthermore, a recent study concluded that advertising of sweet and fruit flavored e-cigarettes may increase the appeal of e-cigarettes and be associated with less knowledge of e-cigarette health risks among youth [41]. Furthermore, peer use of e-cigarettes, which facilitates the social acceptance norm of vaping, may also contribute to youth’s ease of use perceptions. Nevertheless, we were unable to control for youth’s exposure to e-cigarette marketing and their peer use of e-cigarettes due to a lack of data from the wave 1 youth survey of the PATH Study. Therefore, future studies are warranted to understand whether and how e-cigarette advertising and peer use may explain or predict youth’s ease perceptions about flavored e-cigarette use.

Our analyses also revealed that females and racial and ethnic minorities (i.e., NH Blacks and Hispanics) were more likely than males and NH Whites, respectively, to perceive flavored e-cigarettes as easier to use. This finding is congruent with the high prevalence of flavored tobacco product use in females and racial and ethnic minority groups, partly due to the tobacco industry’s targeted marketing of flavored tobacco products [42]. Our results suggest that such targeted marketing may have led to these groups’ perceptions of the ease of using flavored e-cigarettes, although more research is needed to confirm these hypothesized associations. Our study also showed that younger youth (12–14 years) were more likely than their older peers (15–17 years) to report “I don’t know” to the ease of use question. This finding suggests that knowledge and perceptions in regards to e-cigarettes may be forming in the middle stages of adolescence (around 14–15 years of age) and that e-cigarette preventive efforts are particularly needed to target youth of this age period to curb the development of pro-e-cigarette perceptions.

### Strengths and limitations

This study has three major strengths. First, we used a prospective longitudinal design to assess e-cigarette use behaviors within a one-year follow-up period after controlling for important demographic and psychosocial covariates. Second, the study sample was restricted to never users of tobacco products, avoiding potential confounding between perceiving flavored e-cigarettes as easier to use and the effects of having a history of tobacco use. Third, instead of collapsing responses, we examined each level of the perception related to ease of use, including “I don’t know,” which permitted a more nuanced analysis of this variable.

This study has some limitations. First, youth may perceive the “flavored e-cigarettes easier to use” question differently: some may consider “easier to use” as related to the taste (e.g., sweetness, harshness, and bitterness) of e-cigarette flavors, while others may understand it in terms of the sophisticated techniques and tricks (e.g., cloud chasing) [43] involved in vaping flavored e-liquids. Future studies should conduct cognitive testing to understand how youth perceive this question in order to better interpret the study results. Second, past-30-day e-cigarette use (defined as past-30-day e-cigarette use) may not indicate regular or lasting e-cigarette use among youth. Past research has shown that youth who used e-cigarettes in the past 30 days are mostly infrequent e-cigarette users [44]. Although any form of e-cigarette use among youth never tobacco users would impose negative health impacts, future research may also investigate whether perceived ease of using flavored e-cigarettes is associated with a higher frequency and/or longer duration of e-cigarette use, a behavior that may bring lasting, detrimental health effects to the youth inexperienced tobacco users. Additionally, studies have shown that there is a possibility for youth to recant their lifetime substance use including tobacco products [45,46]. This could imply that the youth who reported having initiated e-cigarette use at wave 2 could recant ever e-cigarette use in the future assessment. However, we are not able to conduct any sensitivity analysis to address this issue since, once the respondents report ever tobacco use in the previous waves, they will not be asked about their ever use of the products again in the upcoming waves. Future studies should examine and consider e-cigarette use recanting and its implications among youth. Lastly, the influence of the rapid-changing landscape of e-cigarette marketplace on e-cigarette use behavior may not have been captured by this study. With the recently increased popularity of emerging e-cigarette brands, such as JUUL, e-cigarette use in youth might have increased after the wave 2 data of the PATH study were collected in 2014–2015. Future studies are needed to assess the role of perceptions related to e-liquid flavors on pod-devices like JUUL.

### Study implications

Given the negative consequences of perceiving flavored e-cigarettes as easier to use as well as the negative health impact of e-cigarette use among youth never tobacco users, public health prevention efforts and regulatory actions should focus on reducing and eliminating the factors that form youth’s perceived ease of using flavored e-cigarettes. First, educating youth never tobacco users about the harms and risks associated with flavored e-cigarettes (e.g., nicotine addiction, respiratory health problems, and openness to cigarette smoking, etc.) could help reduce the perceived benefits of using flavored e-cigarettes among this group. Prevention messages can disclose the tobacco industry’s efforts to allure youth, females, and racial and ethnic minority groups by manufacturing flavored tobacco with improved flavor and taste. Second, the FDA and local tobacco control agencies can consider restrictions of flavored e-cigarette advertising and promotion through banning alluring messages about the taste of e-cigarette flavors as well as regulating labeling and packaging of flavored e-cigarettes with food and cartoon images [47]. These initiatives may reduce e-cigarette flavors’ relatability and attractiveness to youth and potentially diminish youth’s perceived ease of using flavored e-cigarettes. Lastly, the FDA and local policymakers may consider limiting the variety of child-friendly flavors such as fruit and candy flavors or restricting the sale of e-cigarettes with non-tobacco flavors. These legislative actions may substantially reduce youth’s availability to flavored e-cigarettes, thereby making it more difficult for youth to access flavored e-cigarette products.

## Abbreviations

E-cigarettes: Electronic Cigarettes
PATH: The Population Assessment of Tobacco and Health
FDA: The U.S. Food and Drug Administration
ANPRM: The Advance Notice of Proposed Rulemakings
